# Technology Innovations in Dementia Care: Improvements in Functional Assessments Through Background Sensing

**DOI:** 10.1093/geroni/igab046.011

**Published:** 2021-12-17

**Authors:** Matthew Smith, Marcia Ory, Gang Han, Ashley Wilson, Zane Foster, John Fitch

**Affiliations:** 1 Texas A&M School of Public Health, College Station, Texas, United States; 2 Texas A&M University, College Station, Texas, United States; 3 Texas A&M University School of Public Health, College Station, Texas, United States; 4 Texas A&M Center for Population Health and Aging, Texas A&M University School of Public Health, College Station, Texas, United States; 5 Birkeland Current LLC, Waco, Texas, United States

## Abstract

Technological innovations are becoming commonplace in research among persons living with dementia (PLWD) and their caregivers. However, few studies attempt to validate technology’s ability to appropriately monitor functional assessment in dementia care research. Bringing together industry, academia, and health care, we demonstrate the feasibility of using a novel in-situ sensor system to continuously and accurately assess daily functions for PWLDs in home or assisted care settings. Phase1 revealed a high accuracy (~85%) of detecting and classifying ADLs between sensors and human loggers across 26 defined activities. Phase 2, which will target 140 PLWDs, has already demonstrated the value of such sensors in detecting safety concerns (e.g., no heat). Technology-driven research for PLWD and their caregivers have practical applications for assessing diverse forms of functional assessment and environmental conditions which can improve measurement precision over time and space and the ability to better tailor care plans for PLWDs and their caregivers.

